# Postoperative hypernatremia is associated with worse brain injuries on EEG and MRI following pediatric cardiac surgery

**DOI:** 10.3389/fcvm.2023.1320231

**Published:** 2023-12-21

**Authors:** Rouyi Lin, Na Du, Jinqing Feng, Jianbin Li, Xiaowei Li, Yanqin Cui, Shuyao Ning, Mingjie Zhang, Guodong Huang, Huaizhen Wang, Xinxin Chen, Li Ma, Jia Li

**Affiliations:** ^1^ Guangdong Provincial Key Laboratory of Research in Structural Birth Defect Disease, Guangzhou Women and Children’s Medical Center, Guangzhou Medical University, Guangdong, China; ^2^ Clinical Physiology Laboratory, Institute of Pediatrics, Guangzhou Women and Children’s Medical Center, Guangzhou Medical University, Guangdong, China; ^3^ Heart Center, Guangzhou Women and Children’s Medical Center, Guangzhou Medical University, Guangdong, China; ^4^ Department of Electroneurophysiology, Guangzhou Women and Children’s Medical Center, Guangzhou Medical University, Guangzhou, Guangdong Province, China; ^5^ Department of Radiology, Guangzhou Women and Children’s Medical Center, Guangzhou Medical University, Guangzhou, Guangdong Province, China

**Keywords:** congenital heart disease, hypernatremia, EEG, brain injuries, cardiac surgery

## Abstract

**Objectives:**

Dysnatremia is a common electrolyte disturbance after cardiopulmonary bypass (CPB) surgery for congenital heart disease (CHD) and a known risk factor for adverse neurological events and clinical outcomes. The objective of this study was to evaluate the association of dysnatremia with worse abnormal EEG patterns, brain injuries detected by magnetic resonance imaging (MRI) and early adverse outcomes.

**Methods:**

We monitored continuous EEG in 340 children during the initial 48 h following cardiac surgery. Demographics and clinical characteristics were recorded. Sodium concentrations were measured in the arterial blood gas analysis every 6 h. Hyponatremia and hypernatremia were classified by the average of sodium concentrations over 48 h. Postoperative cerebral MRI was performed before hospital discharge.

**Results:**

In our patient cohort, dysnatremia was present in 46 (13.5%) patients. Among them, hyponatremia occurred in 21 (6.2%) and hypernatremia in 25 (7.4%). When compared to patients with normonatremia, hyponatremia was not associated with EEG abnormalities and early adverse outcomes (*Ps *≥ .14). In hypernatremia group, the CPB time was significantly longer and more frequent use of DHCA (*Ps *≤ .049). After adjusting for time, CPB time and the use of DHCA, hypernatremia was significantly associated with worse EEG abnormalities (including background, seizures and pathological delta brushes), more severe brain injuries on MRI (*Ps *≤ .04) and trended to be associated with longer postoperative mechanical ventilation time (*P *= .06).

**Conclusion:**

Hypernatremia and hyponatremia were common in children after cardiac surgery. Hypernatremia, but not hyponatremia, was significantly associated with worse EEG abnormalities and more severe brain injuries on MRI and extended postoperative mechanical ventilation time.

## Introduction

Perioperative brain injuries and long term neurodevelopmental impairment are the prevalent complications in children with congenital heart disease (CHD) undergoing cardiac surgery and are resulted from a combination of demographic and perioperative factors as well as socioeconomic status and genetic factors etc. (1–6). Development of dysnatremia including both hyponatremia and hypernatremia is frequently encountered in children undergoing cardiac surgery (7–11). Recent studies have shown that postoperative hypernatremia is more common than hyponatremia in infants (7, 11), and correlated with increased mortality rate, adverse neurological events and longer hospital stay.

Electrolyte imbalances can impact multiple organs and tissues, with potential repercussions for the central nervous system, notably the brain (12). The nervous system functions in a highly intricate environment. The maintenance of normal neurological function needs precise distribution and concentration of electrolytes (13). Acute hypernatremia results in the hyperosmolality and cellular shrinkage (especially in infants), and consequently it leads to the modification of the synaptic structure and function of the neutrons in the central nervous system (12). Continuous electroencephalographic (EEG) monitoring offers a real-time depiction of the brain's surface electrical activity, thereby presenting a time-sensitive approach for identifying brain injury and dysfunction (3). However, there has been no study using EEG, to the best of our knowledge, to assess brain injury and dysfunction in children with dysnatremia following cardiac surgery. Previously, we comprehensively analyzed perioperative EEG background (including sleep-wake cycling, SWC) and discharge (seizures, spikes/sharp waves and delta brushes) abnormalities in children undergoing cardiac surgery, and found that these EEG background and discharge abnormalities were significantly correlated with the degree of brain injury on MRI and adverse early postoperative clinical outcomes (1). In the present study, we aimed, first, to investigate the association of dysnatremia (hyponatremia and hypernatremia) with EEG abnormalities. The secondary aim was to examine the association betweeen dysnatremia and MRI injuries as well as early adverse outcome.

## Patients and methods

This study entailed a retrospective analysis of data collected prospectively (1).

### Patients

**Figure 1:**
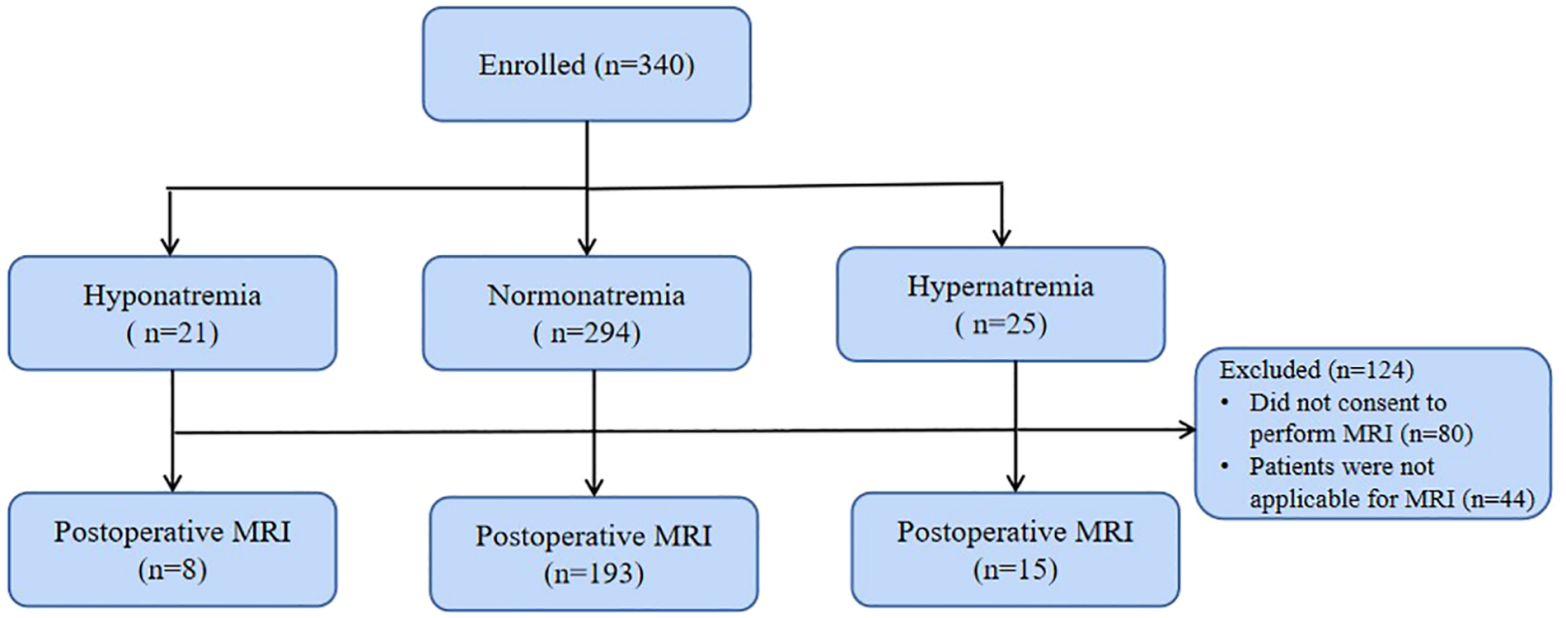
Flow chart of patient populations undergoing EEG and MRI assessment.

The institutional research ethics board approved this study (No. 46201), and informed consent was obtained from parents. A cohort of 340 patients was enrolled between January 2019 and December 2021 (Figure 1) (2, 14). Patients with a recognizable syndrome of congenital anomalies, previous cardiopulmonary bypass (CPB), scalp vein puncture, post-menstrual age <37W, or cerebral hemorrhage by Doppler ultrasound were excluded (1).

### Intraoperative procedures and postoperative managements

Intraoperative procedures and standard postoperative management protocol were used as described elsewhere (1, 2). Primary maintenance fluid was 250 ml of 5% glucose with 7 ml of 10% potassium chloride at an infusion rate of 2–3 ml/kg/h. Continuous infusion of furosemide 0.1–0.4 mg/kg/h was used for control of daily fluid balance in the CICU. Sedation consisted of continuous intravenous infusion of sufentanil (0.2 µg/kg/h) and dexmedetomidine (2 µg/kg/h) and intermittent administration of midazolam (0.1 mg/kg). There was no blood transfusion in the present patient cohort.

### Methods of measurements

#### Sodium concentrations

Sodium concentrations were measured in the arterial blood gas analysis using GEM Premier 3000 (Instrumentation Laboratory, Massachusetts, USA) and recorded every 6 h during the first 48 h after cardiac surgery. The average of sodium values over the 48 h study period was calculated to represent the overall sodium level of each patient. Hypernatremia was defined as average sodium >145 mmol/L and hyponatremia as average sodium <135 mmol/L. Normonatremia was defined as average sodium levels within the range of 135–145 mmol/L. Patients were categorized into hypernatremia, hyponatremia or normonatremia groups based on their postoperative average sodium levels.

#### EEG monitoring

Continuous Video-EEG was recorded using Nicolet monitor (CareFusion, Middleton, Wisconsin, USA) according to the international 10–20 system. The EEG background characteristics were assessed and classified on a scale of 0–4, representing: (1) slow-disorganized; (2) discontinuous; (3) burst-suppression; (4) attenuated-featureless (15). Abnormal SWC was characterized by the absence of SWC in neonates; and the absence of stage 2 transients (K-complexes and spindles) in older children (15, 16). Electrographic seizures was defined as the presence of epileptiform discharges with an average frequency exceeding 2.5 Hz for a duration of at least 10 s. Status epilepticus was characterized by continuous seizures lasting for at least 10 min or accounting for a total duration of at least 20% within any 60-min recording period (17). Spike/sharp waves were characterized by a transient clearly distinguished from background activity (≥2.5 times of background voltage), with pointed peak at a conventional time scale and duration from 20 to 200 ms with duration measured at the EEG baseline (17). Delta brushes comprised of slow waves (0.3–1.5 Hz) with superimposed fast activity (8–22 Hz) (16). Qualified technicians (RYL and SYN) independently analyzed all EEGs in 3-h segments and the final review was conducted by SYN.

#### Cerebral MRI

The MRI scans was conducted using a 3 T Magentom Prisma scanner (Siemens, Munich, Germany). The imaging protocol encompassed standard T1, T2, diffusion-weighted imaging, and diffusion-tensor imaging. MRI scans were reviewed for brain injuries (hemorrhage, white matter injury, and stroke) and were graded according to a standard method into categories of mild, moderate, and severe (18). All MRI scans were reviewed by a neuroradiologist (MJZ). Patients (*n* = 44) with cardiac pacemakers, metal implants or with severe medical conditions were considered not applicable for brain MRI scans.

#### Clinical data

We collected demographic data, STS-EACTS Mortality Category, the postoperative mechanical ventilation time, the uses and durations of deep hypothermic circulatory arrest (DHCA) and CPB, CICU and hospital stay, and death. Early adverse outcomes were defined as prolonged postoperative mechanical ventilation time (>72 h), CICU, hospital stay and death.

### Statistical analysis

Values were expressed as median (range) or frequency (%) where appropriate. Comparisons of parameters across the three groups were made using Kruskal–Wallis test for non-normal distribution variables and Chi-squared or Fish's exact test for categorical variables when appropriate. Post hoc test for multiple comparisons was made using Bonferroni correction. Mixed linear regression analysis for repeated measures (background degree, the number of spikes/sharp wave and delta brush) was used to determine the nature of any time trend over the 48-h study period. It was also used to compare the differences in levels between groups. Binary variables (lack of return to normal background or normal SWC, occurrence of seizures or delta brush, white matter injuries, strokes, hemorrhage and death) were analyzed by logistic regression analysis. The outcomes were evaluated respectively for hyponatremia vs. normonatremia and for hypernatremia vs. normonatremia. The parameter estimates indicated the degree or slope and direction of the correlations. Statistical significance was determined at a probability value of <0.05 (SAS 9.4, Cary, NC, the USA).

## Results

### Patient characteristics

**Figure 2:**
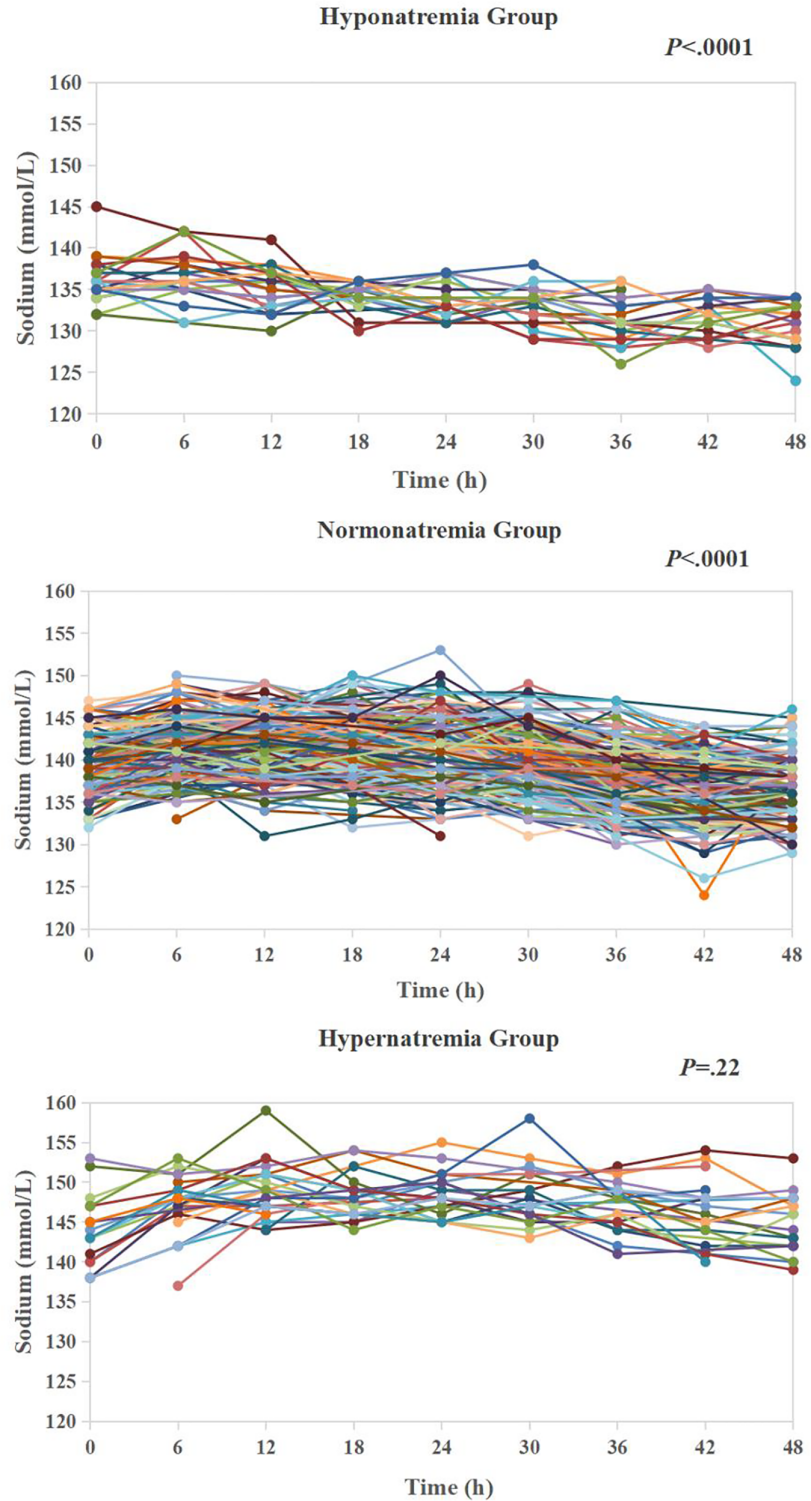
Temporal trends of sodium in hyponatremia group (*n* = 21), normonatremia group (*n* = 294) and hypernatremia (*n* = 25) during 48 h after cardiac surgery.

Table 1 presents demographic and clinical data of 340 patients. CPB was performed in 321 (94.4%) patients (duration ranged 28–338 min, median 111). DHCA was employed in 57 (16.8%) patients (duration ranged 13–42 min, median 18). Non-CPB surgery was performed in 19 (5.6%) patients to repair the coarctation of the aorta or patent ductus arteriosus closure. Three patients (0.9%) had delayed sternal closure. Two (0.6%) patients were treated with peritoneal dialysis during 48 h after cardiac surgery, and one (0.3%) patient was treated with ECMO on postoperative day 4. Six patients (1.8%) died after cardiac surgery. Four of them died of cardiac failure, and two died of respiratory failure. The overall level of sodium concentrations in the entire cohort decreased significantly over the 48 h after surgery (parameter estimate −0.11; *P *< .0001). When all the sampling time points of the cohort were pooled together, the sodium concentrations ranged 124–159 mmol/L (median 140). Hyponatremia (average sodium level ranged 132.2–134.9 mmol/L, median 134.0) hypernatremia (average sodium level ranged 145.1–151.5 mmol/L, median 146.4) groups consisted of 21 (6.2%) and 25 (7.4%) patients respectively, leaving 294 (86.5%) patients in normonatremia group (average sodium ranged 135–145 mmol/L, median 139.2) in our patient cohort. During the first 48 h, the sodium gradually and significantly decreased in hyponatremia and normonatremia groups (parameter estimate −0.13; *P *< .0001 and parameter estimate −0.12; *P *< .0001, respectively), but did not change significantly in hypernatremia group (parameter estimate −0.02; *P *= .22) (Figure 2) (Supplementary Table S1).

**Table 1:** Demographic and clinical data in 340 patients undergoing cardiac surgery.

Variable	Number (%) or median (range)
Gender	
Male, *n* (%)	204 (60)
Female, *n* (%)	136 (40)
Age (day)	107 (1–859)
Weight (kg)	5 (2–11)
BSA (m2)	0.28 (0.16–0.51)
STS-EACTS mortality category, *n* (%)	
1	91 (26.8)
ASD repair, patch	5
VSD repair, patch	58
TOF repair, no ventriculotomy	28
2	118 (34.7)
Coarctation of aorta repair	20
TOF repair, ventriculotomy, transannular patch	45
RVOT procedure	14
PDA closure, surgical	10
VSD, multiple, repair	4
Valvuloplasty, tricuspid	16
Bidirectional cavopulmonary anastomosis	1
Cor triatriatum repair	1
Valvuloplasty, mitral	1
Valvuloplasty, pulmonic	3
PA origin from ascending aorta repair	2
Anomalous origin of coronary artery repair	1
3	74 (21.8)
ASO	6
RV to PA conduit placement	6
Rastelli	1
Pulmonary atresia with VSD repair	3
PA sling repair	4
Coarctation of aorta and VSD repair	43
Complete AVSD repair	8
REV surgery	1
ASD partial closure	2
4	57 (16.8)
Modified B-T shunt for pulmonary atresia and VSD	3
Unifocalization of MAPCAs for pulmonary atresia and VSD closure	3
ASO and VSD repair	11
TAPVC repair	13
IAA repair	14
PA banding	3
DORV, intraventricular tunnel repair	6
Valve replacement, mitral	1
Ross–Konno procedure	1
Pulmonary venous stenosis repair	2
Non-CPB surgery, *n* (%)	19 (5.6)
CPB, *n* (%)	321 (94.4)
CPB time (min)	111 (28–338)
DHCA, *n* (%)	57 (16.8)
DHCA time (min)	18 (13–42)
Hyponatremia, *n* (%)	21 (6.2)
Normonatremia, *n* (%)	294 (86.5)
Hypernatremia, *n* (%)	25 (7.4)
Postoperative mechanical ventilation time (h)	46 (0–1,218)
Delayed sternal closure	3 (0.9)
CICU stay (day)	5 (0–159)
Hospital stay (day)	12 (4–164)
ECMO, *n* (%)	1 (0.3)
Peritoneal dialysis, *n* (%)	2 (0.6)
Death, *n* (%)	6 (1.8)

BSA, body surface area; STS-EACTS, The Society of Thoracic Surgeons-European Association for Cardio-Thoracic Surgery; ASD, atrial septal defect; VSD, ventricular septal defect; TOF, tetralogy of Fallot; RVOT, right ventricular outflow tract; PDA, patent ductus arteriosus; PA, pulmonary artery; MAPCA, major aortopulmonary collateral artery; RV, right ventricle; MAPCA, major aortopulmonary collateral artery; TAPVC, total anomalous pulmonary venous connection; IAA, interrupted aortic arch; DORV, double outlet right ventricle; CPB, cardiopulmonary bypass; DHCA, deep hypothermic cardiac arrest; CICU, cardiac intensive care unit.

### Postoperative EEG abnormalities and brain injuries on MRI

EEG background abnormalities were found in 170 (50.0%) patients, and the degree of abnormalities was significantly lessened over 48 h (parameter estimate −0.004; *P *< .0001). Among them, 89 (26.2%) patients did not recover to normal background by the 48th hour after surgery. Abnormal SWC was found in 51 (15.0%) patients during 48 h leaving 20 (5.9%) patients who did not recover to normal by the 48th hour after surgery. EEG seizures was found in 29 (8.5%) patients. Seizure onset time ranged 0–45 h (median 24) after surgery and the duration of seizures lasted 1.6–1,980.1 min (median 75.62). Among them, 22 (75.9%) patients had status epilepticus. Spikes/sharp waves was found in 255 (75.0%) patients with a significant decrease in their number within 48 h (parameter estimate −0.45; *P *< .0001). Pathological delta brushes was found in 7 (2.1%) patients in young infants (postmenstrual age ranged 37–39 week, median = 38) (Table 2 and Supplementary Table S1).

**Table 2:** Occurrences of postoperative brain injuries on EEG and MRI in hyponatremia, normonatremia and hypernatremia groups.

Variable	Entire cohort (*n* = 340)	Hyponatremia (*n* = 21)	Normonatremia (*n* = 294)	Hypernatremia (*n* = 25)
EEG abnormalities				
Degree of background abnormalities, *n* (%)	170 (50.0)	12 (57.1)	145 (49.3)	13 (52.0)
Seizures, *n* (%)	29 (8.5)	2 (9.5)	21 (7.1)	6 (24.0)
Spikes/sharp waves, *n* (%)	255 (75.0)	15 (71.4)	221 (75.2)	19 (76.0)
Delta brushes, *n* (%)	7 (2.1)	0 (0)	6 (2.0)	1 (4.0)
Abnormal sleep-wake cycle, *n* (%)	51 (15.0)	2 (9.5)	42 (14.3)	7 (28.0)
Lack of return to normal background (%)	89 (26.2)	4 (19.0)	73 (24.8)	12 (48.0)
Lack of return to normal SWC (%)	20 (5.9)	0 (0)	18 (6.1)	2 (8.0)
Brain injury on MRI	*n* = 216	*n* = 8	*n* = 193	*n* = 15
Types of brain injury				
White matter injury, *n* (%)	13 (6.0)	0 (0)	9 (4.7)	4 (26.7)
Stroke, *n* (%)	6 (2.8)	0 (0)	6 (3.1)	0 (0)
Hemorrhage *n* (%)	120 (55.6)	1 (12.5)	105 (54.4)	14 (93.3)
Degree of brain injury on MRI				
Normal, *n* (%)	87 (40.3)	7 (87.5)	79 (40.9)	1 (6.7)
Mild, *n* (%)	114 (52.8)	1 (12.5)	103 (53.4)	10 (66.7)
Moderate, *n* (%)	6 (2.8)	0 (0)	5 (2.6)	1 (6.7)
Severe, *n* (%)	9 (4.1)	0 (0)	6 (3.1)	3 (20.0)

MRI, magnetic resonance imaging.

Postoperative MRI was undertaken in 216 (63.5%) patients at the median 9 (3–37) days postoperatively. Hemorrhage was found in 120 (55.6%) patients presenting subdural in 115, intraparenchymal/intraventricular in 5. White matter injury was identified in 13 (6.0%) patients, and strokes in 6 (2.8%) patients. Overall, 129 (59.7%) patients had brain injury. Among them, 114 were mild, 6 moderate and 9 severe (Table 2).

### Relations of dysnatremia with EEG abnormalities and brain injuries on MRI

When comparing demographic and clinical data of the hyponatremia and hypernatremia groups respectively to the normonatremia group, there were no significant differences in patients' age (*P *= .52), weight (*P *= .83), body surface area (BSA) (*P *= .79), STS-EACTS Mortality Category (*P *= .79), and the DHCA time (*P *= .08). The CPB time was significantly longer (*P *< .0001) and the use of DHCA was more frequent in hypernatremia group (*P *= .049) (Table 3).

**Table 3:** Demographic and perioperative data in hypo-, hyper- and normonatremia groups.

Variable	Hyponatremia	Normonatremia	Hypernatremia	*P*-value
Gender				0.71
Male, *n* (%)	11 (52.4%)	177 (60.2%)	16 (64.0%)	
Female, *n* (%)	10 (47.6%)	117 (39.8%)	9 (36.0%)	
Age (day)	120 (11–859)	109 (1–850)	61 (5–422)	0.52
Weight (kg)	4.7 (3.0–11.0)	5.1 (2.0–10.8)	4.7 (2.3–9.5)	0.83
BSA (m2)	0.28 (0.20–0.51)	0.29 (0.16–0.51)	0.27 (0.16–0.45)	0.79
STS-EACTS mortality category, *n* (%)				0.79
1	6 (28.6%)	81 (27.6%)	4 (16.0%)	
2	7 (33.3%)	100 (34.0%)	11 (44.0%)	
3	3 (14.3%)	65 (22.1%)	6 (24.0%)	
4	5 (23.8%)	48 (16.3%)	4 (16.0%)	
CPB time (min)	92 (28–220)	108 (34–338)	162 (43–289)*^#^	<.0001
DHCA, *n* (%)	1 (4.8%)	48 (16.3%)	8 (32.0%)*^#^	0.049
DHCA time (min)	29 (29–29)	19 (14–40)	16 (13–42)	0.08

BSA, body surface area; STS-EACTS, The Society of Thoracic Surgeons-European Association for Cardio-Thoracic Surgery; CPB, cardiopulmonary bypass; DHCA, deep hypothermic cardiac arrest.

* Compared with hyponatremia group, *P *< 0.05.

^#^ Compared with normonatremia group, *P *< 0.05.

*P*-values < 0.05 were underlined.

The assessment of the EEG abnormalities and early outcomes in hyponatremia group relative to normonatremia group was made after adjusting for time, and showed that hyponatremia was not significantly associated with EEG abnormalities (including the degree of background abnormalities, lack of return to normal background and SWC, occurrence and duration of seizures, the number of spikes/sharp waves, the number and presence of pathological delta brushes) (*Ps *≥ .16) and early adverse outcomes (including postoperative mechanical ventilation time, CICU and hospital stay, as well as death) (*Ps *≥ .14).

The assessment of the EEG abnormalities and early outcomes in hypernatremia group relative to normonatremia group was made after adjusting for time, CPB time and the use of DHCA, and showed that hypernatremia was significantly and positively associated with the degree of background abnormalities (*P = *.008), the incidence of lack of return to normal background by the 48th hour after surgery (*P = *.03), occurrence of seizures (*P = *.04), MRI degree of brain injuries (*P = *.001) including the incidence of white matter injuries (*P *= .02) and hemorrhage (*P *= .04), as well as trend to be positively associated with postoperative mechanical ventilation time (*P = *.06). Pathological delta brushes in normonatremia group occurred in 6 patients (number ranged 2–52, median = 12) and in hypernatremia group in 1 patient (number range 4–107, median = 26). Hypernatremia was not significantly associated with the incidence of pathological delta brushes (*P *= .51) but significantly associated with their number over 48 h (*P = *.009). Hypernatremia was not associated with the lack of return to normal SWC, duration of seizures, the number of spikes/sharp waves, stroke, CICU, hospital stay and death (*Ps *≥ .14) (Table 4).

**Table 4:** Postoperative EEG abnormalities and MRI degree of brain injury and early outcomes in patients with hyponatremia, normonatremia and hypernatremia.

	Hyponatremia vs. normonatremia			Hypernatremia vs. normonatremia^a^		
Variable	Parameter estimate	95% CI	*P*-value	Parameter estimate	95% CI	*P*-value
Degree of background abnormalities^b^	−0.007	−0.28 to 0.27	0.96	0.37	0.10–0.65	0.008
Lack of return to normal background	0.33	−0.78 to 1.46	0.56	0.97	0.11–1.84	0.03
Lack of return to normal SWC	12.17	−722.30 to 746.0	0.97	0.47	−1.12 to 2.06	0.56
Occurrence of seizures	−0.31	−1.84 to1.21	0.69	1.10	0.04–2.17	0.04
Duration of seizures	500.71	−2,630.28 to 3,631.7	0.74	1,574.83	−531.79 to 3,681.46	0.14
Number of spikes/sharp waves^b^	−17.40	−41.76 to 6.98	0.16	7.83	−13.96 to 29.60	0.48
Presence of delta brushes	11.10	−751.50 to 773.70	0.98	0.78	−1.55 to 3.12	0.51
Number of delta brushes^b^	0.17	−0.46 to 0.79	0.59	1.22	0.31–2.13	0.009
MRI degree	0.55	0.08–1.03	0.02	0.62	0.24–0.99	0.001
White matter injury	11.12	−795.10 to 817.40	0.98	1.67	0.22–3.08	0.02
Stroke	11.09	−971.80 to 994.0	0.98	−11.32	−713.20 to 690.60	0.97
Hemorrhage	2.16	0.04–4.27	0.046	2.21	0.14–4.27	0.04
Mechanical ventilation time	13.06	−34.12 to 60.25	0.59	45.39	−1.18 to 91.95	0.06
CICU stay	0.19	−4.89 to 5.29	0.94	1.90	−2.89 to 6.70	0.44
Hospital stay	−4.33	−10.03 to 1.36	0.14	3.39	−1.87 to 8.67	0.21
Death	11.09	−823.50 to 845.70	0.98	0.83	−1.47 to 3.14	0.48

SWC, sleep-wake cycling; MRI, magnetic resonance imaging; CICU, cardiac intensive care unit.

^a^ Variables analyses were adjusted for cardiopulmonary bypass time and the use of deep hypothermic cardiac arrest.

^b^ Variables analyses were adjusted for time.

*P*-values < 0.05 were underlined.

## Discussion

To our knowledge, this is the first study that links dysnatremia with brain injuries on EEG in children with CHD after cardiac surgery. In our patient cohort, the overall dysnatremia in the first 48 h after surgery was present in 46 (13.5%) patients. Among them, hyponatremia occurred in 21 (6.2%) and hypernatremia in 25 (7.4%). Hypernatremia, but not hyponatremia, was significantly associated with worse EEG abnormalities including background, seizures and pathological delta brushes. Moreover, patients with hypernatremia were more likely to have severe brain injuries on MRI and longer postoperative mechanical ventilation time.

The occurrence of dysnatremia in our cohort (13.5%) was relatively low. In previous studies, postoperative abnormal sodium balance in children after cardiac surgery occurred in 47%–65% of patients within postoperative 48 or 72 h (7, 9–11). It should be noted that we defined dysnatremia based on the average sodium level during the postoperative 48 h whereas previous studies were based on some time points (the highest value or lowest value at 0, 24, 48 or 72 h after surgery) (7–11). We considered that our definition may represent a more persistent dysnatremia status over the postoperative period which may reveal a clearer and greater impact on the EEG abnormalities and clinical outcomes. In some previous studies, the incidence of hypernatremia was substantially higher than that of hyponatremia (7, 11). In our patients, the incidences were about the same, in consistency with other study (9).

Generally, hyponatremia has gained more attention in post-cardiac surgery patients due to its association with volume overload, and its potential adverse impact on both cardiac and respiratory functions (11). Moreover, low serum sodium can contribute to the emergence and intensity of cerebral edema and intracranial hypertension. Several studies have shown its association with early adverse outcomes (9, 19–21). But this was not found in our study. When comparing hyponatremia and normonatremia groups, there were no significant differences in brain injuries detected by EEG and early outcomes. One study reported that mild-moderate hyponatremia (sodium levels ranged between 125 and 135 mmol/L) was not associated with adverse outcomes, in consistency with our findings (11). The sodium levels in our hyponatremia group were mostly within this range.

CPB may change serum sodium concentration and extracellular fluid osmolality. Initially, hyponatremia occurred after the commencement of CPB. By the end of CPB, sodium concentration increased above preoperative level (8). Overcorrection of hyponatremia has been found to be associated with prolonged hospitalization and increased mortality rate (8). Furthermore, metabolic acidosis is common and postoperative correction of metabolic acidosis with NaHCO_3_ may result in hypernatremia (22). It is also known that postoperative systemic inflammatory response can induce endothelial damage and vascular hyperpermeability, making the prevention of electrolyte disturbances and fluid overload complicated (10, 23). More recent studies have reported that hypernatremia, but not hyponatremia, was associated with worse postoperative neurologic and clinical outcomes (longer mechanical ventilation time, hospital stay and more death) (7, 11). The neurologic outcomes were assessed using clinical seizures and stroke (7). These findings are supported by our present study. Our data showed that hypernatremia, but not hyponatremia, was associated EEG abnormalities and adverse clinical outcomes. In our study, patients with hypernatremia had prolonged CPB duration and more frequent use of DHCA, similar to previous reports (8, 10, 11). Importantly, after adjusting of CPB time and the use of DHCA, the correlations of hypernatremia with EEG abnormalities (including background, seizures and pathological delta brushes) remained significant.

Among the EEG abnormalities, background abnormalities occurred in half of the patient populations in our present study and other studies (3, 5). Background abnormalities reflect abnormal brain maturation or/and basic brain injury. A rabbit experiment has shown that hypernatremia produced by saline loading induced abnormal EEG background (24). The disorder of sodium induces central nervous system depression and may cause diffused brain dysfunction that may be attributable to the background abnormalities (12, 25). Part of the background abnormalities is the lack of return to normal SWC. We previously reported that the incidence of lack of return to normal SWC by 48 h after cardiac surgery was associated with early adverse outcomes (1). The presence study, did not show any significant association of hypernatremia with abnormal SWC. The reason remains unclear.

EEG seizures occurred in 8.5% in our patients. Hypernatremia was associated with higher incidence of seizures compared to normonatremia group (24% vs. 7%), being consistent with previous studies (7, 24). The potential mechanisms for this finding might be speculated. Firstly, changes in electrolyte gradients across cellular membranes have both direct and indirect impacts, influencing neuronal excitability, synchronization, and potentially promoting epileptiform activities (26). Secondly, hypernatremia induced brain shrinkage can lead to the rupture of cerebral veins, resulting in focal intracerebral and subarachnoid hemorrhages, which in turn may trigger seizures (12).

Few studies have examined pathological delta brushes in children following cardiac surgery. The presence of a delta brush pattern in preterm infants serves as an indicator for assessing brain maturation and activity (27). Pathological delta brushes refer to those occurring in full term babies. It has been reported that plasma hyperosmolality in neonatal rats stimulates only a subset of the osmoresponsive brain regions in adult rats suggesting that the immature brain is more poorly regulated for hyperosmolality (28). Our data further showed that hypernatremia was not significantly associated with the number of spikes/sharp waves. The spikes/sharp waves indicate subtle impairments in cerebral microstructure and neuronal networks, which is more likely to be induced by limited oxygen and energy supply (29). Its relation with dysnatremia remains to be explored.

In general, neurologic symptoms associated with electrolyte disorders are primarily functional rather than structural. MRI assessment showed brain structural injuries before hospital discharge. We found that hypernatremia was associated with the MRI degree of brain injuries and almost all (93.3%) patients with hypernatremia had subdural hemorrhage. The primary consequence of hypernatremia is the shrinkage of cerebral cells that resulting in the brain being pulled away from the overlying calvarium, leading to the tearing of bridging veins and the occurrence of extensive subdural hemorrhage (30, 31). Our data further showed that hypernatremia was associated with increase occurrence of white matter injury. In a previous study on severe hypernatremia (serum sodium >160 mmol/L) in infants showed the incidence of white matter injuries was up to 70% (31). Experimental data in rats have demonstrated that acute hypernatremia can result in enduring histological brain damage, which includes myelinolysis of the white matter and neuronal necrosis (32).

## Limitations

Our study has several limitations: (1) It has inherent limitations due to the observational study design. The associations found in this study cannot clarify any causal relationship. (2) We did not accurately record the precise amounts of intravenous crystalloids, sodium bicarbonate, and enteral or parenteral nutrition, as these are sources of exogenous sodium and water with substantial variability. (3) The influence of hypernatremia on neurodevelopmental outcomes has yet to be investigated.

## Future direction

Further research is warranted to clarify the causal relationship between hypernatremia and early brain injury and long-term neurodevelopmental outcomes in randomized trials comparing patients with controlled normonatremia and those under conventional treatment.

## Conclusion

Hypernatremia was common in our patient cohort and significantly associated with worse EEG abnormalities (including background, seizures and pathological delta brushes) and more severe brain injuries on MRI and longer postoperative mechanical ventilation time. In clinical practice, more attention should be paid to maintain normonatremia in children after cardiac surgery.

## Data Availability

The raw data supporting the conclusions of this article will be made available by the authors, without undue reservation.
