# Supplementary material for: Postoperative hypernatremia is associated with worse brain injuries on EEG and MRI following pediatric cardiac surgery
Source: Front Cardiovasc Med. 2023 Dec 21;10:1320231. doi: 10.3389/fcvm.2023.1320231 (PMC10768027; doi:10.3389/fcvm.2023.1320231)
Supplement: Supplementary file 1 [file Table1.docx]

**Supplemental Table 1.** Postoperative trends of sodium, EEG background and discharge abnormalities among the three groups.

| **CICU time** (hours) | **Sodium (mmol/L)**  Mean (SD) | **Degree of background abnormalities** Mean (SD) | **Number of patients with Seizures**  Number | **Spikes/sharp waves (times/h)** Median (range) | **Delta brushes (times/h)** Median (range) |
| --- | --- | --- | --- | --- | --- |
| **Hyponatremia group (n=21)** | | | | | |
| 0-3 | 136.2±2.8 | 0.6±0.5 |  | 17.0 (1.0-270.3) | 0 (0-0) |
| 3-6 |  | 0.5±0.5 |  | 18.5 (1.3-260.0) | 0 (0-0) |
| 6-9 | 137.0±3.0 | 0.6±0.5 |  | 11.0 (0-238.0) | 0 (0-0) |
| 9-12 |  | 0.5±0.5 |  | 8.2 (0.7-200.0) | 0 (0-0) |
| 12-15 | 135.0±2.6 | 0.5±0.5 |  | 14.0 (1.7-172.7) | 0 (0-0) |
| 15-18 |  | 0.4±0.5 |  | 13.3 (1.0-432.6) | 0 (0-0) |
| 18-21 | 134.3±1.6 | 0.4±0.5 |  | 30.0 (1.5-271.7) | 0 (0-0) |
| 21-24 |  | 0.5±0.8 |  | 15.0 (1.3-507.5) | 0 (0-0) |
| 24-27 | 133.7±2.2 | 0.4±0.5 | 1 | 11.3 (0-355.0) | 0 (0-0) |
| 27-30 |  | 0.4±0.5 | 1 | 9.7 (0-206.7) | 0 (0-0) |
| 30-33 | 133.1±2.4 | 0.3±0.5 |  | 10.0 (0-290.0) | 0 (0-0) |
| 33-36 |  | 0.3±0.5 |  | 8.3 (0-518.3) | 0 (0-0) |
| 36-39 | 131.3±2.7 | 0.2±0.4 |  | 10.5 (0-136.7) | 0 (0-0) |
| 39-42 |  | 0.1±0.4 |  | 8.3 (2.5-165.0) | 0 (0-0) |
| 42-45 | 131.7±2.2 | 0.1±0.3 |  | 10.3 (0-160) | 0 (0-0) |
| 45-48 | 130.8±2.7 | 0.1±0.4 |  | 10.0 (0-37.5) | 0 (0-0) |
| Statistical analysis results | | | | | |
| Parameter estimate | -0.13 | -0.009 |  | -0.56 | / |
| P value | <0.0001 | <0.0001 |  | 0.01 | / |
| **Normonatremia group (n=294)** | | | | | |
| 0-3 | 139.8±3.0 | 0.6±0.8 | 2 | 10.0 (0-680.7) | 0 (0-0) |
| 3-6 |  | 0.5±0.7 | 1 | 9.0 (0-1075.0) | 0 (0-0) |
| 6-9 | 142.1±3.0 | 0.5±0.7 |  | 10.2 (0-923.3) | 0 (0-0) |
| 9-12 |  | 0.5±0.7 |  | 9.0 (0-808.7) | 0 (0-10.0) |
| 12-15 | 141.4±3.3 | 0.5±0.7 | 1 | 8.7 (0-421.3) | 0 (0-26.7) |
| 15-18 |  | 0.4±0.6 | 1 | 8.8 (0-265.0) | 0 (0-51.7) |
| 18-21 | 141.5±3.6 | 0.4±0.6 | 2 | 12.0 (0-438.3) | 0 (0-40.0) |
| 21-24 |  | 0.3±0.7 |  | 13.7 (0-550.0) | 0 (0-15.7) |
| 24-27 | 140.4±3.6 | 0.4±0.7 | 2 | 14.6 (0-356.7) | 0 (0-16.0) |
| 27-30 |  | 0.4±0.7 |  | 13.2 (0-408.3) | 0 (0-33.3) |
| 30-33 | 138.9±3.4 | 0.4±0.7 | 5 | 13.0 (0-600.0) | 0 (0-48.3) |
| 33-36 |  | 0.4±0.7 | 3 | 12.0 (0-403.3) | 0 (0-36.7) |
| 36-39 | 137.6±3.6 | 0.4±0.8 | 1 | 11.7 (0-304.7) | 0 (0-16.7) |
| 39-42 |  | 0.4±0.8 | 2 | 11.3 (0-303.3) | 0 (0-21.0) |
| 42-45 | 136.6±3.9 | 0.5±0.9 |  | 13.3 (0-536.7) | 0 (0-41.7) |
| 45-48 | 136.6±3.4 | 0.5±0.9 | 1 | 10.5 (0-320.0) | 0 (0-23.3) |
| Statistical analysis results | | | | | |
| Parameter estimate | -0.12 | -0.005 |  | -0.41 | 0.008 |
| P value | <0.0001 | <0.0001 |  | <0.0001 | 0.0003 |
| **Hypernatremia group (n=25)** | | | | | |
| 0-3 | 143.8±4.6 | 0.6±0.9 | 1 | 16.5 (0-364.0) | 0 (0-9.5) |
| 3-6 |  | 0.8±1.1 |  | 15.0 (0-355.0) | 0 (0-18.3) |
| 6-9 | 147.1±3.8 | 1.1±1.4 | 1 | 11.5 (0-356.7) | 0 (0-21.5) |
| 9-12 |  | 1.0±1.3 |  | 14.5 (0-271.7) | 0 (0-18.3) |
| 12-15 | 148.8±3.5 | 0.8±1.2 |  | 10.6 (0-413.3) | 0 (0-46.7) |
| 15-18 |  | 0.8±1.1 |  | 10.0 (0-450.0) | 0 (0-30.0) |
| 18-21 | 148.4±2.9 | 0.9±1.3 |  | 21.8 (2.0-345.0) | 0 (0-0) |
| 21-24 |  | 0.9±1.3 | 2 | 27.2 (0-293.3) | 0 (0-10.0) |
| 24-27 | 148.3±2.8 | 0.9±1.3 | 2 | 27.5 (1.3-226.7) | 0 (0-3.3) |
| 27-30 |  | 0.8±1.3 |  | 17.7 (0-258.3) | 0 (0-51.7) |
| 30-33 | 148.2±3.6 | 0.7±1.2 |  | 19.0 (1.0-241.7) | 0 (0-60.0) |
| 33-36 |  | 0.7±1.2 |  | 15.5 (0-198.3) | 0 (0-106.7) |
| 36-39 | 146.8±3.0 | 0.8±1.2 |  | 9.4 (0-345.0) | 0 (0-47.5) |
| 39-42 |  | 0.9±1.2 |  | 21.0 (0-395.0) | 0 (0-43.3) |
| 42-45 | 146.0±4.3 | 1.2±1.4 |  | 16.7 (1.0-240.0) | 0 (0-6.7) |
| 45-48 | 145.0±3.7 | 1.3±1.5 |  | 24.3 (0.7-500.0) | 0 (0-0) |
| Statistical analysis results | | | | | |
| Parameter estimate | -0.02 | 0.005 |  | -0.77 | 0.03 |
| P value | 0.22 | 0.06 |  | 0.003 | 0.34 |

Note. *CICU* = cardiac intensive care unit.
